# *ABO* gene polymorphisms are associated with acute coronary syndrome and with plasma concentration of HDL-cholesterol and triglycerides

**DOI:** 10.17305/bb.2023.9244

**Published:** 2023-12-01

**Authors:** Gilberto Vargas-Alarcón, Oscar Pérez-Méndez, Rosalinda Posadas-Sánchez, Héctor González-Pacheco, Alexandra Arias-Mendoza, Galileo Escobedo, Teresa Juárez-Cedillo, Marva Arellano-González, José Manuel Fragoso

**Affiliations:** 1 Departamento de Biología Molecular, Instituto Nacional de Cardiología Ignacio Chávez, Mexico City, México; 2 Departamento de Endocrinología, Instituto Nacional de Cardiología Ignacio Chávez, Mexico City, México; 3 Unidad Coronaria, Instituto Nacional de Cardiología Ignacio Chávez, Mexico City, México; 4 Unidad de Medicina Experimental, Hospital General de Mexico, Dr. Eduardo Liceaga, Mexico City, México; 5 Unidad de Investigación en Epidemiologia y Servicios de Salud-Área de Envejecimiento. Centro Médico Nacional Siglo XXI. Instituto Mexicano del Seguro Social, Mexico City, México

**Keywords:** Polymorphisms, acute coronary syndrome (ACS), human ABO blood group system

## Abstract

The role of *ABO* gene polymorphisms in acute coronary syndrome (ACS) and lipid metabolism is increasingly recognized. We investigated whether *ABO* gene polymorphisms are significantly associated with ACS and the plasma lipid profile. Six *ABO* gene polymorphisms (rs651007 *T/C*, rs579459 *T/C*, rs495928 *T/C*, rs8176746 *T/G*, rs8176740 *A/T*, and rs512770 *T/C*) were determined by 5’exonuclease TaqMan assays in 611 patients with ACS and 676 healthy controls. The results demonstrated that the rs8176746 *T* allele was associated with a lower risk of ACS under the co-dominant, dominant, recessive, over-dominant, and additive models (*P* ═ 0.0004, *P* ═ 0.0002, *P* ═ 0.039, *P* ═ 0.0009, and *P* ═ 0.0001, respectively). Furthermore, under co-dominant, dominant, and additive models, the rs8176740 *A* allele was associated with a lower risk of ACS (*P* ═ 0.041, *P* ═ 0.022, and *P* ═ 0.039, respectively). On the other hand, the rs579459 *C* allele was associated with a lower risk of ACS under the dominant, over-dominant, and additive models (*P* ═ 0.025, *P* ═ 0.035, and *P* ═ 0.037, respectively). In a subanalysis performed with the control group, rs8176746 *T* and rs8176740 *A* alleles were associated with low systolic blood pressure and with both high high-density lipoprotein-cholesterol (HDL-C) and low triglyceride plasma concentrations, respectively. In conclusion, *ABO* gene polymorphisms were associated with a lower risk of ACS, and lower systolic blood pressure and plasma lipid levels, suggesting a causal relationship between ABO blood groups and the incidence of ACS.

## Introduction

Acute coronary syndrome (ACS) is characterized by a partial or total thrombotic obstruction of coronary artery caused by the rupture or erosion of atherosclerotic plaque. ACS is a set of clinical complications, including unstable angina and myocardial infarction with or without ST-segment elevation [1, 2]. This syndrome is multifactorial, resulting from the combination of genetic background and cardiovascular risk factors, such as obesity, dyslipidemia, and hypertension, among others, that play an important role in the development of atherosclerotic plaque [1–4]. In the last years, the association between ABO blood groups and plasma lipid levels has shown that carriers of non-O type had higher levels of total cholesterol, and low-density lipoprotein cholesterol (LDL-C), leading to early development of cardiovascular diseases [5–7]. The ABO blood groups are encoded by the ABO gene located at q34.2 of chromosome 9. Recent studies have established the association of six genetic variants of the *ABO* gene with a high prevalence of different cardiovascular diseases, including ACS, in different populations, the rs579459 *T/C*, rs8176746 *T/G*, rs512770 *T/C*, rs495928 *T/C*, rs651007 *T/C*, and rs8176740 *A/T* [7–17]. However, the association of *ABO* gene polymorphisms with ACS is still controversial and merits to be further explored.

Considering the potential impact of ABO groups on cardiovascular diseases, we hypothesized that the *ABO* gene polymorphisms are associated with the incidence of ACS, and with plasma lipid profile in the Mexican population. To explore this possibility, the present study aimed to establish whether the *ABO* rs651007 *T/C*, rs579459 *T/C,* rs495928 *T/C,* rs8176746 T/G, rs8176740 *A/T*, and rs512770 *T/C* polymorphisms are associated with the risk of developing ACS. In addition, we evaluated whether these polymorphisms are associated with plasma lipid profile.

**Table 1:** Information of the studied polymorphism tested

**Gene^a^** **symbol**	**dbSNP^a^**	**Chromosome (NCBI Build 156)^a^**	**Position (NCBI Build 156)^a^**	**Change base (pb)**	**Location in gene^a^**	**Tagged ABO blood group**
*ABO*	rs651007	9q34.2	133278431	*T > C*	5’-UTR	A
*ABO*	rs579459	9q34.2	133278724	*C > T*	5’-UTR	A
*ABO*	rs495828	9q34.2	133279294	*T > G*	5’-UTR	O
*ABO*	rs8176746	9q34.2	133255935	*T > G*	Exon 7	B
*ABO*	rs8176740	9q34.2	133256085	*T > A*	Exon 7	A
*ABO*	rs512770	9q34.2	133258116	*T > C*	Exon 5	O

^a^The chromosomal location for each SNP table was obtained by querying each SNP “rs” number in the NCBI single nucleotide polymorphism database (dbSNP), build 156 on the 21st of September 2022 (http://www.ncbi.nlm.nih.gov/projects/SNP/).

## Materials and methods

### Characteristics of the study population

We included 1287 Mexican mestizo individuals, 611 patients with ACS, and 676 healthy controls. The sample size was calculated for an unmatched case-control study with a power of 80% and an alpha error of 0.05 (http://www.openepi.com/SampleSize/SSCC.html). From July 2015 to May 2018, 611 patients with ACS (81.5% men and 18.5% women, with a mean age of 57.7 ± 9.9 years) were referred to the Instituto Nacional de Cardiología Ignacio Chávez. The diagnosis of ACS was made following the European Society of Cardiology (ESC) and American College of Cardiology (ACC) definitions [18, 19]. Clinical characteristics, electrocardiographic changes, and biochemical markers (creatine phosphokinase isoenzymes, troponin I) were determined for diagnosis. The control group included 676 healthy individuals (66.4% men and 33.5% women, with a mean age of 54.0 ± 7.7 years) that were recruited from the cohort of Genetics of Atherosclerosis Disease (GEA) Mexican study database [20]. Control subjects were asymptomatic and apparently healthy without clinical or family history of premature coronary artery disease (CAD), congestive heart failure, liver, renal, thyroid, or oncological disease. In addition, control subjects had a coronary calcium score of zero determined by computed tomography, indicating the absence of subclinical atherosclerosis in these individuals [20]. The association of *ABO* gene polymorphisms with plasma lipid levels was evaluated only in controls. All the included subjects were ethnically matched and considered Mexican mestizos only if they and their ancestors (at last three generations) were born in Mexico.

### Laboratory analyses

Cholesterol and triglyceride plasma concentrations were determined by enzymatic/colorimetric assays (Randox Laboratories, Crumlin, UK). High-density lipoprotein-cholesterol (HDL-C) plasma concentrations were determined by the phosphotungstic acid-Mg^2+^ method. LDL-C concentrations were calculated in samples with triglyceride concentrations lower than 400 mg/dL using Friedewald’s formula [21]. Dyslipidemia was defined as the presence of one or more of the following conditions: cholesterol > 200 mg/dL, LDL-C > 130 mg/dL, HDL-C < 40 mg/dL, or triglycerides > 150 mg/dL, according to the guidelines of the National Cholesterol Education Project (NCEP) Adult Treatment Panel (ATP III) (https://www.nhlbi.nih.gov/resources/third-report-expert-panel-detection-evaluation-and-treatment-high-blood-cholesterol-0 [accessed on May 2, 2023]). According to the MSD manual guidelines, type 2 diabetes mellitus (T2DM) was considered when participants had a fasting glucose level ≥ 126 mg/dL, previously diagnosed by a physician (https://www.msdmanuals.com/professional/endocrine-and-metabolic-disorders/diabetes-mellitus-and-disorders-of-carbohydrate-metabolism/diabetes-mellitus-dm∖#v29299021 [accessed on May 2, 2023]). Hypertension was defined by a systolic blood pressure ≥ 140 mmHg, diastolic blood pressure ≥ 90 mmHg, or the use of oral antihypertensive therapy.

### Genetic analysis

DNA extraction was performed from total blood as previously described [22]. The determination of the *ABO* 5’UTR rs651007 *T/C*, *ABO* 5’UTR rs579459 *T/C,* *ABO* 5’UTR rs495928 *T/C,* *ABO* Leu266Met rs8176746 *T/G*, *ABO* Phe216Ile rs8176740 *A/T*, and *ABO* Pro74Ser rs512770 *T/C* polymorphisms were performed using 5’exonuclease TaqMan assays on a 7900HT Fast Real-Time PCR system in accordance with manufacturer’s instructions (Applied Biosystems, Foster City, USA) (Table 1). As a quality control, 10% of the samples were genotyped twice; results were concordant for all cases.

### Ethical statement

The study was conducted in accordance with the Declaration of Helsinki and approved by the Ethics and Research Committees of Instituto Nacional de Cardiología Ignacio Chávez (protocol number: 22-1288, approved: 08/February/2022). Informed consent was obtained from all subjects involved in the study.

**Table 2:** Demographic, clinical, and biochemical parameters of the studied individuals

**Characteristics**		**ACS patients (*n* ═ 611)**	**Healthy controls (*n* ═ 676)**	***P*-value**
Age (years)		57.7 ± 9.9	54.3 ± 7.6	<0.001
Gender, *n* (%)	Male	498 (81.5)	449 (66.4)	<0.001
	Female	113 (18.5)	227 (33.5)	
BMI (kg/m^2^)		27 [25–29]	28 [26–31]	0.09
Blood pressure (mmHg)	Systolic	130 [114–144]	115 [106–126]	<0.001
	Diastolic	80 [70–90]	72 [66–77]	<0.001
Glucose (mg/dL)		127 [101–188]	91 [84–99]	<0.001
Total cholesterol (mg/dL)		163 [127–198]	188 [164–210]	<0.001
HDL-C (mg/dL)		37 [32–45]	42 [35–52]	<0.001
LDL-C (mg/dL)		103 [75–132]	115 [94–134]	<0.001
Triglycerides (mg/dL)		148 [109–199]	152 [112–209]	0.166
Hypertension, *n* (%)	Yes	345 (56)	202 (30)	<0.001
Type 2 diabetes mellitus, *n* (%)	Yes	214 (35)	64 (9)	<0.001
Dyslipidemia, *n* (%)	Yes	522 (85)	481 (71)	<0.001
Smoking, *n* (%)	Yes	216 (35)	149 (22)	<0.001

Data are expressed as median or interquartile interval [25^th^–75^th^], *n* (% of total patients), or mean ± SD. *P* values were estimated using Student’s *t*-test or Mann–Whitney *U*-test for continuous variables and chi-square test for categorical values. BMI: Body mass index; HDL-C: High-density lipoprotein-cholesterol; LDL-C: Low-density lipoprotein-cholesterol; ACS: Acute coronary syndrome.

### Statistical analysis

Gene frequencies of *ABO* polymorphisms in patients and controls were obtained by direct counting. The Hardy–Weinberg equilibrium was evaluated in patients with ACS and individual controls by chi-squared test. Data analysis was performed with SPSS program version 18.0 (IBM, Chicago, IL). Either Mann–Whitney U or Student’s *t*-test was performed to compare the continuous variables. For categorical variables, chi-squared or Fisher’s exact test was used. The analysis association of the rs651007 *T/C*, rs579459 *T/C*, rs495928 *T/C*, rs8176746 *T/G,* rs8176740 *A/T*, and rs512770 *T/C* single nucleotide polymorphisms (SNPs) with the risk of ACS was performed under the following inheritance models: additive, which compares the subgroup of homozygotes subjects carrying the major allele with heterozygotes as well as with the minor allele homozygotes; codominant, which compares the subgroup of homozygous individuals carrying the major allele with homozygotes of the minor allele; dominant, which compares the subgroup of homozygous individuals carrying the minor allele with the subgroup conformed by heterozygotes and major allele homozygotes; heterozygous, which compares the subgroup conformed by homozygotes carrying major allele and homozygotes carrying minor allele vs heterozygotes; recessive, which compares the subgroup conformed by heterozygotes and major allele homozygotes vs homozygotes of the minor allele. These analyses were performed by a logistic regression, adjusted for cardiovascular risk factors, and determined whether the presence of the genetic variant is associated with the occurrence of the disease [23, 24]. The Bonferroni test was used to correct the *P* values (*P*) according to the number of tests per SNP based on the different models of inheritance. The values of *P* < 0.05 were considered statistically significant, and all odds ratios (OR) were presented as 95% confidence intervals. For the subset of controls grouped by genotypes, the plasma lipid concentrations were expressed as means ± SD, and comparisons were performed by ANOVA and F-test. *P* values < 0.05 were considered statistically significant. The statistical power to detect an association of the polymorphisms with ACS was 0.80 according to the OpenEpi software (http://www.openepi.com/SampleSize/SSCC.html [accessed on 17 June 2021]).

Linkage disequilibrium analysis (LD, D’) and haplotype design were performed using Haploview version 4.1 (Broad Institute of Massachusetts Institute of Technology and Harvard University, Cambridge, MA, USA). This software analyzes the combination of alleles in a single gene, or alleles in multiple genes along a chromosome that tend to be inherited together due to their chromosomal proximity, providing the statistical calculation of LD, D’, logarithm of the odds (LOD) and r-squared, as well as possible haplotype patterns from primary genotypes, using the Human Haplotype Map project data [25].

## Results

### Characteristics of the study sample

Demographic, clinical, and biochemical parameters of the ACS patients and healthy controls are presented in Table 2. There were significant differences between the ACS patients and healthy controls. As expected, traditional risk factors of cardiovascular diseases were altered in ACS patients; glucose plasma levels and blood pressure, frequency of hypertension, dyslipidemia, and smoking habit were higher in patients than in controls. Conversely, the total cholesterol, and LDL-C concentrations in ACS patients were lower than those in the control group; this effect is associated to their anti-dyslipidemic treatment, and changes of lifestyle after the clinical event.

**Table 3:** Association of *ABO* polymorphisms with ACS accordance to the inheritance models

**Polymorphic site (rsID-number)**	**Model**	**Genotype**	**ACS patient, *n* ═ 611 (*n*[%])**	**Controls, *n* ═ 676 (*n*[%])**	**OR (95%CI)**	***P-*value**
rs8176746	Co-dominant	*GG* *GT* *TT*	540 (0.884) 69 (0.113) 2 (0.003)	555 (0.821) 112 (0.166) 9 (0.013)	0.17 (0.03–0.97)	**0.0004**
	Dominant	*GG* *GT + TT*	540 (0.884) 71 (0.116)	555 (0.821) 121 (0.179)	0.49 (0.34–0.72)	**0.0002**
	Recessive	*GG + GT* *TT*	609 (0.997) 2 (0.003)	667 (0.987) 9 (0.013)	0.19 (0.03–1.06)	**0.039**
	Over-dominant	*GG + TT* *GT*	542 (0.887) 69 (0.113)	564 (0.834) 112 (0.166)	0.53 (0.36–0.77)	**0.0009**
	Additive	–	–	–	0.50 (0.35–0.71)	**0.0001**
rs8176740	Co-dominant	*TT* *TA* *AA*	180 (0.294) 296 (0.484) 135 (0.221)	167 (0.247) 332 (0.492) 176 (0.261)	0.69 (0.49–1.00)	**0.041**
	Dominant	*TT* *TA + AA*	180 (0.295) 431 (0.705)	167 (0.247) 508 (0.753)	0.71 (0.53–0.95)	**0.022**
	Recessive	*TT + TA* *AA*	476 (0.779) 135 (0.221)	499 (0.739) 176 (0.261)	0.90 (0.65–1.21)	0.479
	Over-dominant	*TT + AA* *TA*	315 (0.515) 296 (0.485)	343 (0.508) 332 (0.492)	0.83 (0.64–1.07)	0.149
	Additive	–	–	–	0.84 (0.70–1.00)	**0.039**
rs579459	Co-dominant	*TT* *TC* *CC*	438 (0.717) 157 (0.257) 16 (0.026)	445 (0.659) 200 (0.296) 30 (0.044)	0.75 (0.53–0.97)	0.081
	Dominant	*TT* *TC + CC*	438 (0.717) 173 (0.283)	445 (0.659) 230 (0.341)	0.72 (0.54–0.96)	**0.025**
	Recessive	*TT + TC* *CC*	595 (0.974) 16 (0.026)	645 (0.956) 30 (0.044)	0.82 (0.40–1.71)	0.599
	Over-dominant	*TT + CC* *TC*	454 (0.743) 157 (0.257)	475 (0.704) 200 (0.296)	0.73 (0.54–0.98)	**0.035**
	Additive	*–*	–	–	0.77 (0.60–0.99)	**0.037**

The *P*-values were calculated by the logistic regression analysis, and ORs were adjusted for gender, age, blood pressure, BMI, glucose, total cholesterol, HDL-C, triglycerides, and smoking habit. Bold: *P* < 0.05; ACS: Acute coronary syndrome; OR: Odds ratio; CI: Confidence interval.

### Association of *ABO* polymorphisms with ACS

Allele and genotype frequencies of the polymorphic sites in patients with ACS and controls were in Hardy–Weinberg equilibrium. In the first analysis, the genetic distribution of the rs512770 *T/C,* rs651007 *T/C*, and rs495928 *T/C*, polymorphisms were similar in patients with ACS and healthy controls. Nonetheless, rs8176746 *T/G,* rs8176740 *A/T*, and rs579459 *T/C* genotype frequencies showed significant differences between ACS patients and healthy individuals (*P* < 0.05) (Table S1). In addition, the association analysis showed that under different models, the *T* allele and *TT* genotype of the rs8176746 *T/G* polymorphism were associated with decreased risk of developing ACS (OR ═ 0.17, 95%CI: 0.03–0.97, *P*_Co-dominant_ ═ 0.0004, OR ═ 0.49, 95%CI: 0.34–0.72, *P*_Dominant_ ═ 0.0002, OR ═ 0.19, 95%CI: 0.03–1.06, *P*_Recessive_ ═ 0.039, OR ═ 0.53, 95%CI: 0.36–0.77, *P*_Over-dominant_ ═ 0.0009, and OR ═ 0.50, 95%CI: 0.35–0.71, *P*_Additive_ ═ 0.0001, respectively). In addition, the *A* allele and *AA* genotype of the rs8176740 *A/T* polymorphism was associated with a lower risk of developing ACS (OR ═ 0.69, 95%CI: 0.49–1.00, *P*_Co-dominant_ ═ 0.041, OR ═ 0.71, 95%CI: 0.53–0.95, *P*_Dominant_ ═ 0.022, and OR ═ 0.84, 95%CI: 0.70–1.00, *P*_Additive_ ═ 0.039, respectively). Finally, the *C* allele and *CC* genotype of the rs579459 *T/C* polymorphism were associated with decreased risk of developing ACS (OR ═ 0.72, 95%CI: 0.54–0.96, *P*_Dominant_ ═ 0.025, OR ═ 0.73, 95%CI: 0.54–0.98, *P*_Over-dominant_ ═ 0.035, and OR ═ 0.77, 95%CI: 0.60–0.99, *P*_Additive_ ═ 0.037, respectively) (Table 3). All models were adjusted for gender, age, blood pressure, BMI, glucose, total cholesterol, HDL-C, triglycerides, and smoking habit. Associations of *ABO* genotypes with ACS remained essentially the same according to the genetic models when data were analyzed without adjustment by traditional risk factors (Table S2).

### Linkage disequilibrium analysis

**Figure 1.**
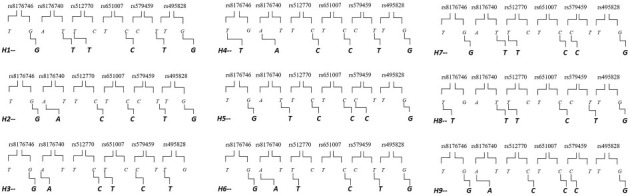
**Scheme depicting the combination of alleles that integrates the nine haplotypes designed by Haploview based on human Haplotype Map project data.** The nomenclature used in the manuscript, H1–H9, is indicated at the bottom of each haplotype.

There was a moderate linkage disequilibrium (D’ ≥ 0.80) between the rs8176746 *T/G*, rs8176740 *A/T*, rs512770 *T/C,* rs651007 *T/C*, rs579459 *T/C,* and rs495928 *T/C* polymorphisms. In addition, this analysis showed six of nine haplotypes (*H1-GTTCTG, H5-GTCCTG, H6-GATCTG, H7-GTTCCG, H8-TTTCTG,* and *H9-GACCCG*, Figure 1) with important differences between the two groups. (Table 4). The H1 and H5 haplotypes were associated with a higher risk of developing ACS (OR ═ 1.24, 95%CI: 1.06–1.44, *pC* ═ 0.007, and OR ═ 3.73, 95%CI: 1.83–7.6, *pC* ═ 0.001, respectively), whereas the H6, H7, H8, and H9 haplotypes were associated with a low risk of developing ACS (OR ═ 0.18, 95%CI: 0.07–0.43, *pC* ═ 0.0001, OR ═ 0.23, 95%CI: 0.10–0.52, *pC* ═ 0.0001, OR ═ 0.16, 95%CI: 0.06–0.48, *pC* ═ 0.0001, and OR ═ 0.26, 95%CI: 0.10–0.69, *pC* ═ 0.0037, respectively).

**Table 4:** Distribution of *ABO* haplotypes in ACS patients and healthy controls

**Polymorphic site (rsID-number)**	**ACS patients, *n* ═ 608**	**Healthy controls, *n* ═ 675**	**OR**	**95%CI**	***P-*value**
rs8176746 *T/G*–rs8176740 *A/T*–rs512770 *T/C–*rs651007 *T/C–*rs579459 *C/T*–rs495828 *T/G*	Hf	Hf			
*H1* (*G T T C T G*)	0.486	0.433	1.24	1.06–1.44	**0.007**
*H2* (*G A C C T G*)	0.260	0.255	1.03	0.85–1.22	0.769
*H3* (*G A C T C T*)	0.129	0.121	1.07	0.85–1.36	0.538
*H4* (*T A C C T G*)	0.048	0.060	0.79	0.56–1.12	0.190
*H5* (*G T C C C G*)	0.028	0.008	3.73	1.83–7.6	**0.0001**
*H6* (*G A T C T G*)	0.005	0.027	0.18	0.07–0.43	**0.0001**
*H7* (*G T T C C G*)	0.006	0.025	0.23	0.10–0.52	**0.0001**
*H8* (*T T T C T G*)	0.003	0.020	0.16	0.06–0.48	**0.0001**
*H9* (*G A C C C G*)	0.004	0.016	0.26	0.10–0.69	**0.0037**

The polymorphism order of haplotypes is according to the position in the chromosome. (rs8176746–rs8176740–rs512770–rs651007–rs579459–rs495828 chromosome 9q34.2). Bold: *P* < 0.05; Hf: Haplotype frequency.

**Table 5:** Distribution of plasma lipid concentrations according to the different genotypes in healthy control group (*n* ═ 676)

***ABO***	***rs8176746 T/G***	* *		
	***GG (*n* ═ 555)***	***GT (*n* ═ 112)***	***TT (*n* ═ 9)***	P-value*
*Parameters*				
BMI (kg/m^2^)	28.17 [25.6–30.7]	27.5 [25.7–29.9]	30.8 [27.7–31.6]	0.115
Blood pressure (mmHg)				
Systolic	115 [106–125]	120.5 [109–129]	110 [105–116]	**0.034**
Diastolic	72 [66–77]	73.5 [68.5–79.5]	70 [67–75.5]	0.489
Glucose (mg/dL)	90 [84–98]	93.5 [85–102]	87 [84–108]	0.666
Total cholesterol (mg/dL)	187.9 [164–210]	188 [166–207]	186 [166–200]	0.504
HDL-C (mg/dL)	42.9 [35.1–53.6]	41 [35.1–48.9]	39.7 [34.5–41.2]	0.255
LDL-C (mg/dL)	115 [93.7–134.2]	114.5 [93.7–131.6]	117 [97.6–133]	0.856
Triglycerides (mg/dL)	151 [112–207]	154 [111–225]	161 [122.4–203.6]	0.837
***ABO***	***rs8176740 A/T***	* *	* *	
	***TT (n ═ 167)***	***TA (n ═ 332)***	***AA (n ═ 176)***	***P-value***
*Parameters*				
BMI (kg/m^2^)	27.7 [25–30.8]	28 [25.7–30.7]	27.7 [25–30.8]	0.292
Blood pressure (mmHg)				
Systolic	115 [107–124]	115 [105–127]	116.5 [108–127]	0.604
Diastolic	72 [66.7–76.5]	72 [66–77.5]	72.5 [66.8–78]	0.438
Glucose (mg/dL)	91 [84–99]	91 [84–99.2]	90 [84–98]	0.521
Total cholesterol (mg/dL)	185 [163.3–205]	190 [165–213]	189 [165.6–207]	0.499
HDL-C (mg/dL)	40 [34.1–50.0]	41.5 [34.6–53.5]	45 [37.2–53.3]	**0.036**
LDL-C (mg/dL)	113 [93.5–131.6]	117 [93.8–136.3]	115 [94.3–132.4]	0.898
Triglycerides (mg/dL)	171 [117.7–208.8]	155 [112.6–223]	138 [107–186.5]	**0.007**
***ABO***	***rs579459 T/C***	* *		
	***TT (n ═ 445)***	***TC (n ═ 200)***	***CC (n ═ 30)***	***P-value***
*Parameters*				
BMI (kg/m^2^)	27.9 [25.8–30.5]	28.2 [25.4–30.7]	27.8 [24.2–31.6]	0.955
Blood pressure (mmHg)				
Systolic	115 [106–126]	116.5 [107–128]	114 [105–124]	0.429
Diastolic	72 [66.5–77]	73 [67–78]	68 [62–76]	0.157
Glucose (mg/dL)	90 [84–99]	92 [84–99.2]	86 [83–93]	0.382
Total cholesterol (mg/dL)	187 [163–207.6]	193 [165.9–215]	196 [176.8–215]	0.087
HDL-C (mg/dL)	42 [35.2–52.1]	42 [34.9–53]	42 [38.8–57]	0.368
LDL-C (mg/dL)	112 [92.9–131.7]	119 [99.6–136.9]	124 [93–139]	0.424
Triglycerides (mg/dL)	151 [112.8–205.6]	154 [113–223.3]	155 [95.5–182]	0.627

Data of BMI, blood pressure, glucose, total cholesterol, HDL-C, LDL-C, and triglycerides are expressed as mean ± SD. Bold: *P* < 0.05; *: ANOVA test; BMI: Body mass index; HDL-C: High-density lipoprotein-cholesterol; LDL-C: Low-density lipoprotein-cholesterol.

### *ABO* polymorphisms and plasma lipids concentrations

Previous reports have suggested that the ABO blood groups are associated with plasma lipid levels [5–7]. To estimate the potential effect of rs8176746 *T/G*, rs8176740 *A/T*, and rs579459 *T/C* polymorphisms, we compared plasma lipids concentrations (total cholesterol, LDL-C, HDL-C, and triglycerides), as well as BMI, blood pressure, and glucose in individuals grouped by genotypes of the three studied polymorphisms (Table 5). For this analysis, we only included the healthy controls group; patients were excluded from this subanalysis because their lipid concentrations were altered by the use of anti-dyslipidemic or anti-hypertensive drugs [26–28]. Interestingly, subjects with the rs8176746 *TT* genotype had lower systolic blood pressures (110 [105–116] mmHg) compared with carriers of either *GT* (120.5 [109–129] mmHg) or *GG* genotypes (115 [105–125] mmHg) (*P* ═ 0.034). On the other hand, individuals with rs8176740 *AA* genotype showed higher concentrations of HDL-C (45 [37.2–53.3] mg/dL) than subjects with either *TA* or *TT* genotypes (Table 5). In addition, individuals with rs8176740 *AA* genotype showed lower concentrations of triglycerides (138 [107–186.5] mg/dL) when compared to *TA* (155 [112.6–223] mg/dL) or *TT* genotype (171 [117.7–208.8] mg/dL). The rs579459 *T/C* polymorphism was not associated with total cholesterol, LDL-C, HDL-C, triglycerides, BMI, blood pressure, or glucose (Table 5).

## Discussion

In the present study, we studied six polymorphisms (rs651007 *T/C*, rs579459 *T/C*, rs495928 *T/C*, rs8176746 *T/G,* rs8176740 *A/T*, and rs512770 *T/C*) located in the *ABO* gene that have been previously implicated in plasma lipids concentrations in CAD [7–17]. In our study, we reported that the rs579459 *C,* rs8176746 *T,* and rs8176740 *A* alleles confer a low risk of ACS. We also found that the H1 and H5 haplotypes were associated with a high risk of developing ACS, whereas the H6, H7, H8, and H9 haplotypes were associated with a low risk. Interestingly, the haplotypes H6 to H9 include the rs579459 *C*, rs8176746 *T*, and rs8176740 *A* alleles that conferred low ACS risk. Importantly, the associations of ABO genotypes remained significant even when data were not adjusted by traditional risk factors (Table S2). To the best of our knowledge, our work is one of the few studies that describe the association of these polymorphisms with ACS. The association of the rs579459 *T/C*, rs8176746 *T/G*, and rs8176740 *A/T* polymorphisms with cardiovascular diseases and ACS in different populations is scarce and controversial. In contrast with our findings, Zhao et al. [11] reported that the *CC* genotype of the rs579459 *T/C* increased the risk of CAD and was related to a higher risk of major adverse cardiovascular events in the Chinese population. Wauters et al. [16] reported that the rs579459 *C* allele was associated with recurrent myocardial infarction, and cardiac death in a cohort of patients with ACS. In line with these data, Paquette et al. [7] reported that rs579459 *C* and rs8176746 *T* alleles increased the risk of developing cardiovascular disease in the Caucasian population with familial hypercholesterolemia. In the same way, Groot et al. [17] reported that the rs8176746 *TT* genotype increased the risk of thromboembolic events, and myocardial infarction in the Caucasian population. In addition, Li and Schooling [13] reported that the *T* allele of the rs8176746 *T/G* SNP is associated with the risk of developing some diseases of the circulatory system, such as ischemia heart disease, myocardial infarction, and major coronary heart disease event. In contrast, Gao et al. [14] reported that the rs579459 *T* allele was associated with the highest risk of developing coronary heart disease in Asian population. Even if controversial, our data and previous reports suggest an association of the rs579459 *T/C*, rs8176746 *T/G,* and rs8176740 *A/T* SNPs with cardiovascular events. The potential mechanisms that could explain the statistical association of *ABO* polymorphisms with cardiovascular outcomes may involve the gut microbiota and platelets aggregation [8, 29–34]. First, A, B, and H carbohydrates are expressed in different tissues and may also be secreted as soluble molecules in certain individuals. The absence of N-acetyl galactosamine transferase activity necessary to structure A antigen, alters gut microbiota in pigs [29]. Also, the secretion of soluble A- and B-antigens seems to affect gut microbiota in humans [30–32]. In this context, gut dysbiosis has related to CAD risk factors, such as obesity, diabetes, dyslipidemia, hypertension, uric acid metabolism, and oxidative stress [33]. Therefore, the association of *ABO* polymorphisms with ACS observed in this study may be mediated by microbiota. Second, A, B, and H carbohydrates are expressed on different glycoproteins on platelet surfaces and are also present on glycans of the von Willebrand factor [34]. Consequently, ABO group determinants may affect the thrombotic process during a coronary event.

It should be emphasized that the frequency of these polymorphisms varies according to the ethnic origin of the study populations. Here, we reported that the frequency of the rs579459 *C,* rs8176746 *T,* and rs8176740 *A* alleles in the Mexican population were 19.2%, 9.6%, and 50.6%, respectively (Supplementary Table 1). According to data obtained from National Center for Biotechnology Information (NCBI) (https://www.ensembl.org/index.html [accessed on 17 January 2022]), the distribution of the rs579459 *C* allele in our population, as well as in the Caucasian and Asian populations was similar (19.2%, 21%, and 19%, respectively). Mexican (from Los Angeles) and African individuals showed a low frequency of this allele (14.1%, and 13%, respectively). Concerning the rs8176746 *T* allele, in our study, in individuals from Los Angeles with Mexican ancestry, as well as in Caucasian, the frequency of this polymorphism was low (9.6%, 8%, and 8%, respectively), whereas Asian and African individuals have a higher frequency of this allele (19% and 17%, respectively). Finally, the individuals from Los Angeles with Mexican ancestry, as well as the population in this study, have a low frequency of the rs8176740 *A* allele (59.4 and 50.6%, respectively) compared with Caucasian, Asian, and African individuals (78%, 71%, and 76%, respectively) (https://www.ensembl.org/index.html [accessed on June 02, 2023]). Considering our results and the different distribution of the *ABO* polymorphisms, we propose that additional studies in other populations with different ethnic origins could help to define the true role of these polymorphisms in the risk of developing ACS.

To explore whether ABO genotypes contribute to ACS incidence via plasma lipid levels, we determined the effect of the rs579459 *T/C*, rs8176746 *T/G,* and rs8176740 *A/T* polymorphisms on plasma lipid concentrations and some cardiovascular risk factors. We found that individuals with the rs8176746 *TT* genotype presented low systolic blood pressure, whereas those with the rs8176740 *AA* genotype presented a higher HDL-C plasma concentration, as well as the lowest triglycerides concentrations among the subgroups. As far as we know, this is the first study that showed the relationship of the rs8176740 *A/T* with plasma lipid concentrations in individuals who were not receiving either anti-dyslipidemic or anti-hypertensive drugs; these drugs modify the plasma lipids profile, masking the real impact of *ABO* gene polymorphisms [26–28]. Information concerning the association between ABO blood groups and plasma lipid concentrations is still scarce. For example, Paquette et al. [7] reported that rs579459 *C* and rs8176746 *T* alleles were associated with an increase of total cholesterol, lipoprotein (a), and non-HDL-C levels with a risk of developing hypercholesterolemia. Li and Schooling [13] reported that the *T* allele of the rs8176746 *T/G* is associated with lower plasma levels of ApoB and LDL-C, but with a risk of developing some cardiovascular diseases. By the same token, Groot et al. [17] reported that rs8176746 *T* allele in B group individuals was associated with a lower risk of hypertension compared with O blood group. Therefore, our results and previous studies suggest a link of *ABO* gene polymorphisms with protection against ACS that may be partially related with HDL-C. The association of ABO groups with HDL plasma levels has been previously reported [35–37] presupposing a modification of lipoprotein metabolism but the mechanisms involved are still unknown [35]. Additional studies, such as GWAS, exome sequencing studies, and recently “exome chip” in a larger number of individuals should be undertaken.

For a correct interpretation of the impact of *ABO* gene polymorphisms on plasma lipid levels and blood pressure, it should be considered that control individuals were recruited based on the inclusion criteria to match some characteristics with patients. Consequently, this group does not represent a random sample of the general population [38] and the association of *ABO* polymorphic sites with HDL-C, triglycerides, and blood pressure were biased. Therefore, the ABO genetic impact on secondary outcomes in our study may not be extensive to general population [38] and warrant future studies *ad hoc* to validate such findings and investigate the potential mechanisms.

Finally, we recognize that our study has some other limitations that merit to be pondered; the number of carriers of some polymorphisms and haplotypes was limited. Also, men in the ACS group were almost five times the number of women, and controls were not matched by sex and age. Considering these limitations, the effect of the SNPs on ACS incidence and some CAD risk factors should be cautiously interpreted.

## Conclusions

This study demonstrated that rs579459 *T/C,* rs8176746 *T/G,* and rs8176740 *A/T* polymorphisms of the *ABO* gene and four haplotypes (H6, H7, H8, and H9) were associated with a decreased risk of ACS in Mexicans. In addition, individuals with the rs8176746 *TT* genotype presented low systolic blood pressure levels, whereas individuals with the rs8176740 *AA* genotype presented high HDL-C concentrations and a low triglycerides concentration.

## Supplemental Data

**Table S1:** Allele and genotype frequencies of *ABO* gene polymorphisms in ACS patients and healthy controls

**Polymorphic site (rsID-number)**	**ACS, *n* ═ 611 (*n*[%])**	**Controls, *n* ═ 676 (*n*[%])**	*P
rs651007 *T/C*			
*Allele*			
*C*	1028 (84.5)	1153 (85.2)	NS
*T*	188 (15.4)	199 (14.7)	
*Genotype*			
*CC*	435 (71.5)	488 (72.1)	
*CT*	158 (25.9)	177 (26.1)	NS
*TT*	15 (2.4)	11 (1.6)	
rs579459 *C/T*			
*Allele*			
*T*	1032 (84.4)	1090 (80.7)	0.013
*C*	190 (15.5)	260 (19.2)	
*Genotype*			
*TT*	438 (71.6)	445 (65.9)	
*TC*	156 (25.5)	200 (29.6)	0.026
*CC*	17 (2.8)	30 (4.4)	
rs495828 *T/G*			
*Allele*			
*G*	1036 (84.7)	1153 (85.2)	NS
*T*	186 (15.2)	199 (14.7)	
*Genotype*			
*GG*	441 (72.1)	487 (72.0)	
*GT*	154 (25.2)	179 (26.4)	NS
*TT*	16 (2.6)	10 (1.5)	
rs8176746 *T/G*			
*Allele*			
*G*	1149 (94.0)	1222 (90.3)	0.0006
*T*	73 (5.9)	130 (9.6)	
*Genotype*			
*GG*	540 (88.4)	555 (82.1)	
*GT*	69 (11.3)	112 (16.5)	0.001
*TT*	2 (0.3)	9 (1.3)	
rs8176740 *A/T*			
*Allele*			
*A*	566 (46.3)	684 (50.6)	0.013
*T*	656 (53.7)	666 (49.3)	
*Genotype*			
*AA*	135 (22.0)	176 (26.0)	
*AT*	296 (48.4)	332 (49.1)	0.048
*TT*	180 (29.4)	167 (24.5)	
rs512770 *T/C*			
*Allele*			
*C*	602 (49.2)	650 (48.1)	NS
*T*	620 (50.7)	702 (51.9)	
*Genotype*			
*CC*	157 (25.6)	157 (23.2)	
*CT*	288 (47.1)	336 (49.7)	NS
*TT*	166 (27.1)	183 (27.0)	

Data are shown as *n* and frequency. *Chi-square test; NS: Not significant; ACS: Acute coronary syndrome.

**Table S2:** Association of the *ABO* polymorphisms with ACS in accordance to the inheritance models without adjusted by cardiovascular risk factors

**Polymorphic site (rsID-number)**	**Model**	**Genotype**	**ACS patients, *n* ═ 611 (*n*[%])**	**Controls, *n* ═ 676** **(*n*[%])**	**OR (95%CI)**	***P*-value**
rs8176746	Co-dominant	*GG* *GT* *TT*	540 (0.884) 69 (0.113) 2 (0.003)	555 (0.821) 112 (0.166) 9 (0.013)	0.23 (0.05–1.06)	**0.0024**
	Dominant	*GG* *GT + TT*	540 (0.884) 71 (0.116)	555 (0.821) 121 (0.179)	0.60 (0.44–0.83)	**0.0015**
	Recessive	*GG + GT* *TT*	609 (0.997) 2 (0.003)	667 (0.987) 9 (0.013)	0.24 (0.05–1.13)	**0.041**
	Over-dominant	*GG + TT* *GT*	542 (0.887) 69 (0.113)	564 (0.834) 112 (0.166)	0.64 (0.46–0.88)	**0.0063**
	Additive	*–*	–	–	0.61 (0.45–0.81)	**0.0007**
rs81746740	Co-dominant	*TT* *TA* *AA*	180 (0.294) 296 (0.484) 135 (0.221)	167 (0.247) 332 (0.492) 176 (0.261)	0.71 (0.52–0.97)	**0.045**
	Dominant	*TT* *TA + AA*	180 (0.295) 431 (0.705)	167 (0.247) 508 (0.753)	0.79 (0.62–1.01)	**0.028**
	Recessive	*TT + TA* *AA*	476 (0.779) 135 (0.221)	499 (0.739) 176 (0.261)	0.80 (0.62–1.04)	**0.048**
	Over-dominant	*TT + AA* *TA*	315 (0.515) 296 (0.485)	343 (0.508) 332 (0.492)	0.97 (0.62–1.04)	0.789
	Additive	–	–	–	0.84 (0.72–0.98)	**0.029**
rs579459	Co-dominant	*TT* *TC* *CC*	438 (0.717) 157 (0.257) 16 (0.026)	445 (0.659) 200 (0.296) 30 (0.044)	0.54 (0.29–1.01)	**0.041**
	Dominant	*TT* *TC + CC*	438 (0.717) 173 (0.283)	445 (0.659) 230 (0.341)	0.76 (0.60–0.97)	**0.026**
	Recessive	*TT + TC* *CC*	595 (0.974) 16 (0.026)	645 (0.956) 30 (0.044)	0.58 (0.31–1.07)	0.076
	Over-dominant	*TT + CC* *TC*	454 (0.743) 157 (0.257)	475 (0.704) 200 (0.296)	0.82 (0.64–1.05)	0.059
	Additive	*–*	–	–	0.77 (0.63–0.95)	**0.013**

Bold: *P* < 0.05; ACS: Acute coronary syndrome; OR: Odds ratio; CI: Confidence interval.
